# Moral Attitudes Toward Pharmacologically Assisted Couples Therapy: An Experimental Bioethics Study of Real-World “Love Drugs”

**DOI:** 10.1080/21507740.2024.2402221

**Published:** 2024-10-18

**Authors:** Mey Bahar Buyukbabani, Brian D. Earp, Ivar Hannikainen, Tommaso Barba, Emilian Mihailov, David B. Yaden, Julian Savulescu

**Affiliations:** a University of Oxford; b Yong Loo Lin School of Medicine, National University of Singapore; c University of Granada; d Imperial College London; e University of Bucharest; f Johns Hopkins University School of Medicine

## PRÉCIS

In a recent study, Lantian and colleagues (2024) measured public attitudes toward the use of ‘love drugs’ as introduced through the work of Earp, Savulescu, and their collaborators. Use of a “revolutionary pill” (described as “100% reliable”) to bring about love is seen as less morally acceptable than psychological therapy toward the same end, and this is partly explained by perceptions that the pill-induced love is less authentic. However, the “pill” in question bears little resemblance to the real-world uses of love drugs discussed by Earp and Savulescu, such as MDMA-assisted couples therapy. In this partial replication and extension study, we show that more ecologically valid ‘love drugs’ scenarios lead to much higher ratings of moral acceptability and perceived authenticity of the resulting love.

## INTRODUCTION

Over a series of papers culminating in a book (Earp and Savulescu 2020a), two of us introduced and examined the idea of using “love drugs”—biochemical interventions intended to preserve or enhance the (quality of) love between partners—as a supplement to traditional means of pursuing the same objectives, such as attending couples therapy. As we emphasize in our work, love drugs on this conception are not a matter of science fiction, but already exist, raising urgent scientific and ethical questions that need to be carefully addressed. A key example of such a drug is 3,4-Methylenedioxymethamphetamine (MDMA, known as “ecstasy” or “molly” when used illegally), a substance that has garnered significant attention in recent years as a potentially promising treatment for serious mental health conditions including post-traumatic stress disorder (PTSD), particularly when combined with psychotherapy (but see ICER 2024 for concerns and limitations).

Although most of this research has focused on individual patients or participants undergoing drug-assisted therapy, there is now a small but growing literature on “MDMA-facilitated cognitive-behavioral *conjoint* therapy” for romantic couples dealing with varying levels of relational dissatisfaction or distress (Monson et al. 2020, 1, emphasis added).^1^ In this literature, it is assumed that at least one of the partners undergoing the conjoint therapy will have been diagnosed with PTSD; thus, the intervention is framed as medical “treatment”—albeit, at the couple-level—for a recognized mental health issue that may have profound interpersonal implications.

By contrast, in our “love drugs” work, we consider the possibility of pharmacologically assisted therapy for couples dealing with a range of so-called “ordinary” relational difficulties that may not rise to the level of a clinical diagnosis, yet which may still be significant for people**’**s lives. Moreover, we caution against the needless pathologization of such difficulties as a prerequisite for future potential access; that is, *assuming* adequate safety and efficacy for such purposes is ever demonstrated (Earp and Savulescu 2020b, 116–22). In other words, we argue for an “enhancement” framework focused on identifying couples who would likely benefit from drug-assisted conjoint therapy (following comprehensive screening for abuse, coercive control, and other relevant factors; see Greenstien 2024), *whether or not* either partner suffers from a diagnosable mental health condition (cf. Cornfield et al. 2024).

But how might the public react to such a proposal? In a previous study, we found that the hypothetical use of psilocybin, a psychedelic drug, for individual-level well-being enhancement was generally morally approved by US participants—despite being an instance of non-medical drug use—when described as occurring under relatively safe, legal, supervised conditions, as in the “Oregon model” of drug regulation (Sandbrink et al. 2024). However, little is known about public attitudes toward the analogous use of a drug for purposes of *relationship* enhancement, i.e., where the goal would be to increase such outcomes as *interpersonal* well-being, couple satisfaction, or even the felt quality of love between partners (cf. Colbert & Hughes 2023).

Looking into this issue, Lantian and colleagues (2024) conducted a study on people**’**s moral attitudes toward “love drugs” using a contrastive vignette design. Across two experiments, the vignettes described a character who realizes they no longer love their partner as much as in the early days of the relationship, who then uses either *love drugs* (experimental condition) or *psychological therapy* (control condition) to “rekindle” their romantic feelings. Lantian et al. report that “the use of love drugs designed to strengthen and maintain love in romantic relationships” was seen by participants as “more morally problematic than psychological therapy with the same aim” (albeit still morally acceptable overall, at least in their second study—which used more realistic descriptions), where this was “partially due to the fact that the love resulting from the use of love drugs is perceived as less authentic, intense, and durable.”

We congratulate Lantian and colleagues for breaking new ground in empirically studying lay attitudes toward “love drugs” for what we believe is the first time. However, although they prominently cite the work of Earp and Savulescu as inspiration for their investigation, the manner in which “love drugs” are described in their experiments bears little resemblance to the conception we—Earp and Savulescu—have employed in our work. In what is perhaps the most striking departure from our approach, Lantian et al. (2024) frame the use of love drugs as being *opposed to* psychological therapy, whereas we have argued against such “stand-alone” use, instead asking how “biochemical agents [might be deployed] *in conjunction* with professional psychotherapy, social support, and other established strategies as a way to help people achieve their relationship goals” (Earp and Savulescu 2020a, 12–3, emphasis added).

In response, Lantian et al. might argue that, in real life, there is no guarantee people would use love drugs in the facilitative-adjunctive way we explore in our work, so it is just as well to consider public attitudes toward the potential use of love drugs “on their own” (i.e., as a replacement for, rather than supplement to, psychotherapy or other established strategies). However, even if so, the way the drugs themselves are described in the experiments by Lantian et al. is incompatible with the conception we have used.

In Study 1, for example, the ostensible love drug is described as a “revolutionary treatment, under the form of a pill” that has been “proven 100% reliable” and—evidently directly, that is, without any effort on the part of the user, much less on the part of the couple working together as a pair—“intensifies the feeling of romantic love that one feels for a person.” Thus, following a month of treatment with the revolutionary pill, it is said that, upon leaving the bathroom one morning, the character “suddenly feels madly in love” with their partner again and decides to stay in the relationship.

By contrast, in Earp and Savulescu (2020a), we urge that any real-world love drug, to be plausible and acceptable, neither would nor should work in a deterministic manner to “directly” produce feelings of love (however authentic or inauthentic). Rather, we suggest that love drugs could, in some cases, support a well-considered decision to stay with a non-abusive partner: “Not all by themselves, of course, but in the context of a lot of hard work, plus therapy, whose healing effects such drugs might one day enhance” (81).

Elsewhere in the same work, we explicitly reject the idea of a “100% reliable” intervention: “there are no actual magic potions out there that will instantly transform your emotional life … the most likely scenario for the foreseeable future, even as neuroscience progresses, will be more or less powerful loadings of the dice—not sorcery” (54–5). In other words, to meet even a minimal threshold of realism or moral desirability on our account, a purported love drug could not be perfectly reliable in bringing about its effects. Moreover, there would have to be a significant role for active effort, engagement, and reflection on the part of the user(s), ideally under the guidance of a qualified therapist.

To account for these differences, we conducted a partial replication and extension of Study 2 from the Lantian et al. (2024) paper (we chose Study 2 because the drug condition was revised to be least somewhat more realistic than in Study 1, e.g., by describing the feeling of love as gradually increasing over time, rather than “suddenly” returning). In a between-subjects design using a convenience sample recruited from Prolific (final *N* = 288 UK participants; 146 women, 141 men, 1 non-binary; age range 21–76, *M*_age_ = 41.4, *SD*_age_ = 13.3; Oxford University IRB approval #R80692/RE006), we randomly assigned participants to read one of 3 vignettes (see Table 1 for exact wording), which vary in their degree of *ecological validity* and thus resemblance to the real-world applications of “love drugs” (e.g., MDMA-assisted couples therapy) discussed by Earp and Savulescu.

**Table 1.** Vignettes used in the study.

LantianS2	LantianRealistic	IdealCase
*Paul is 30 years old and is in a relationship with Sophie for about ten years.* *Regrettably, he realizes that he no longer loves Sophie as much as in the early days of their relationship. He hesitates to stay in a relationship with her.* *Paul then goes to a doctor who recommends that he chooses one among several treatments. After having obtained Sophie****’****s consent, Paul chooses to take a revolutionary new treatment, under a pill form, that could improve his romantic condition by facilitating his appreciation of Sophie****’****s qualities.* ***This revolutionary pill (which has been clinically tested and proven 100% reliable) intensifies the feeling of romantic love that we feel for a person***. [Note: see LantianRealistic, next column over, for the changes made to this condition—the original *“*drug*”* condition from Study 2 of Lantian et al. (2024)—required to make the drug description minimally consistent with the *“*love drugs*”* concept/examples employed by Earp and Savulescu (2020a). Changes in bold.] *After one month of treatment during which Paul had the feeling of a gradual increase of his love for Sophie, he feels again, as in the early days, love for his partner with whom he decides to stay*	*Paul is 30 years old and is in a relationship with Sophie for about ten years.* *Regrettably, he realizes that he no longer loves Sophie as much as in the early days of their relationship. He hesitates to stay in a relationship with her.* *Paul then goes to a doctor who recommends that he chooses one among several treatments. After having obtained Sophie* ***’*** *s consent, Paul chooses to take a revolutionary new treatment, under a pill form, that could improve his romantic condition by facilitating his appreciation of Sophie* ***’*** *s qualities.* ***This revolutionary pill has been clinically tested and proven fairly reliable. However, like any intervention, it is not 100% effective. It doesn’t guarantee a successful result. Instead, it’s more of a "nudge"—a matter of probabilities.*** ***Basically, in combination with other factors—like the mindset and motivation of the person taking the pill—it can intensify the feeling of romantic love that we feel for a person.*** *After one month of treatment during which Paul had the feeling of a gradual increase of his love for Sophie, he feels again, as in the early days, love for his partner with whom he decides to stay.*	*Paul and Sophie are 30 years old and have been in a relationship with each other for about ten years. They are married and have two young children.* *Regrettably, they realize that they no longer love each other as much as in the early days of their relationship. They hesitate to stay in a relationship with each other, and have even considered getting a divorce.* *But still, they think, there are reasons to try to make it work. For one thing, there are the kids to think about. And it* ***’*** *s not like they hate each other. Their values are basically in line. They have a lot in common: shared routines, shared finances, shared history. Their lives, their memories, and even their identities are intertwined.* *If they could only find a way to rekindle the flame between them! But so far nothing has worked. Not the couples retreat they went on. Not talk therapy. Not romantic vacations.* *One day, they read about a new study going on at a prestigious university in their town. The researchers are recruiting long-term romantic partners for a study on "pharmacologically-assisted psychotherapy for couples."* *According to the recruitment materials, the study involves taking a* ***low-risk, non-addictive pill as a supplement to couples counseling. Like any intervention, this pill is not 100% effective. It doesn’t guarantee a successful result. Instead, it temporarily fosters greater feelings of empathy and connectedness between partners and enables people to talk more openly and honestly about their emotions and difficulties.*** ***However, once the immediate effects of the pill wear off—usually after a few hours—the couple still has a lot of work to do to apply whatever they’ve learned from the experience to their daily lives.*** ***Paul and Sophie decide to enroll in the study. The study lasts for one month, during which the couple undergoes two separate pill-enhanced therapy sessions, spaced out by a couple of weeks.*** ***As they work through their problems and learn to see each other again with "fresh eyes,"*** *Paul and Sophie feel a gradual increase in their love for one another. After one month of treatment, each of them feels again, as in the early days, love for each other and they decide to stay together.*

The text in bold reflects different descriptions of how the pill works and its reliability between conditions. *LantianS2* precisely replicates the text from the drug condition of Study 2 from Lantian et al. (2024) and is the least realistic of the 3 vignettes. *LantianRealistic* is the same text with the minimal required changes for the “love drug” to be consistent with the more ecologically valid concept and examples employed in our work (e.g., the drug is not “100%” reliable). And *IdealCase* is loosely adapted from a scenario in Earp and Savulescu (2020a, ch. 5) which is described as the “strongest contender” for the most realistic and morally justified use of love drugs. It thus differs significantly from the other two conditions, both by including details of the situation and motivations of the couple (e.g., they have children) and making explicit that the “pill” is meant to be used as an adjunct to couples therapy. Consequently, the vignette is also substantially longer than the other two; this should be kept in mind as a limitation and addressed by better matching of vignette lengths (i.e., word counts) in future studies.

**Figure 1.**
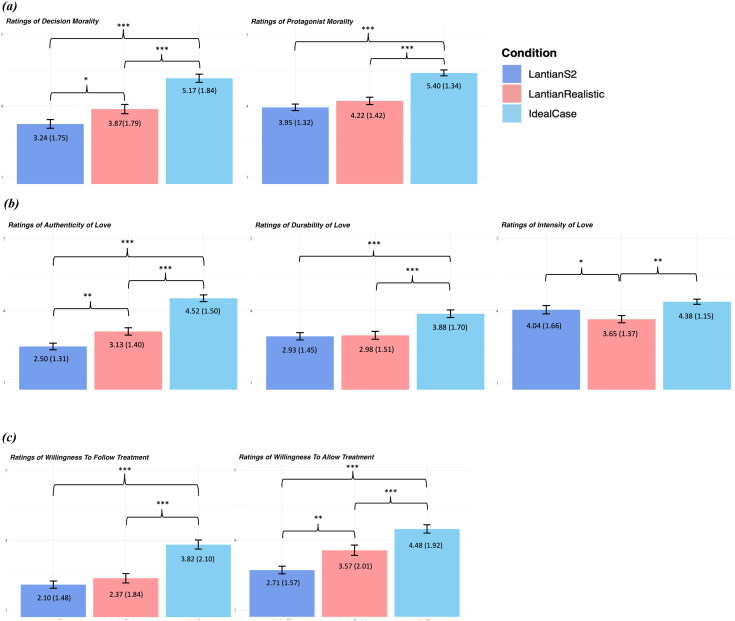
**Study results.** Note: Error bars represent 95% confidence intervals around the mean. The number of asterisks indicates the level of statistical significance: *p < 0.05, **p < 0.01, ***p < 0.001. Means and standard deviations are listed on top of the bars (standard deviation in parenthesis). (a) Mean Scores for the Perceived Morality of Decision/Protagonist by Condition. (b) Mean Scores for Authenticity, Durability, and Intensity of Love by Condition. (c) Mean Scores for Willingness to Follow/Allow Treatment by Condition.

**Figure 2.**
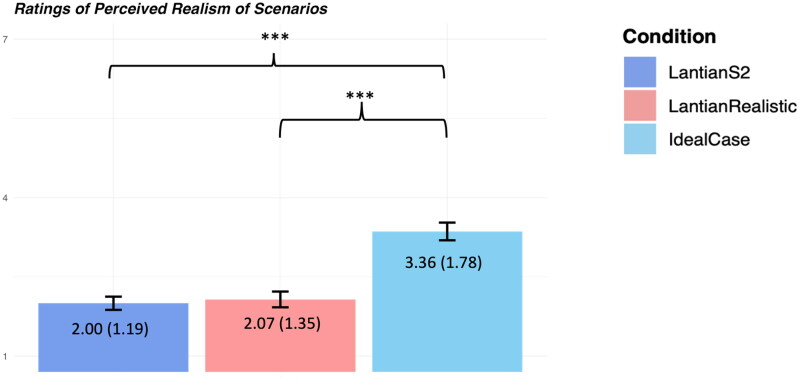
**Perceived realism.** Note: Error bars represent 95% confidence intervals around the mean. The number of asterisks indicates the level of statistical significance: *p < 0.05, **p < 0.01, ***p < 0.001. Means and standard deviations are listed on top of the bars (standard deviation in parenthesis).

We measured the same dependent variables as Lantian et al. (2024). The results are shown in Figure 1. Perceived realism is shown in Figure 2. For complete materials, methods, and detailed statistical analyses, see the Supplemental File and the OSF page for this project at https://osf.io/9na2q/.

## DISCUSSION

In this study, we asked participants to judge an ecologically valid scenario modeled on the realistic prospect of MDMA-assisted couples therapy (*IdealCase*), as well as an unrealistic scenario based on an individual**’**s one-sided, stand-alone use of a “100% reliable” pill described as “revolutionary” (*LantianS2*), replicating Study 2 of Lantian et al. (2024). We found that public attitudes toward the hypothetical use of pharmacology for purposes of relationship enhancement differ dramatically depending on how the case is described (see Lewis et al. 2023 on the need for ecologically valid scenarios in experimental bioethics).

In this UK convenience sample, participants viewed the decision to undergo drug-assisted couples therapy as more morally justified (and the character making the decision as more moral) than a decision to take a “revolutionary pill” to increase feelings of love; they felt that they would be more willing to undergo such therapy themselves, and were more in favor of allowing it than banning it; and they viewed the resulting feeling of love as being much more authentic and durable (though no more intense).

Indeed, even a minimal increase in ecological validity—e.g., stating that the pill was not “100% reliable” but rather that its effects would depend, in part, on the mindset and motivation of the user (*LantianRealistic*)—increased participants**’** moral approval of the decision, their willingness to allow it, and the perceived authenticity of the resulting love. This is striking because, as shown in Figure 2, *LantianRealistic* was not actually judged by participants to be significantly more realistic than *LantianS2*; instead, based on the minimal change to the case that was made, it appears to be the non-deterministic nature of the intervention, allowing room for the user**’**s own will to play a role in rekindling feelings of love, that was responsible for the higher ratings of moral acceptability and authenticity of love observed in Figure 1.

Future work should seek to more closely match (e.g., in word count) and decompose the brute difference in ecological validity between (a) the minimally realistic case we have employed here (*LantianRealistic*) and (b) the “ideal” case of a value-aligned couple with children undergoing drug-assisted couples therapy to preserve their relationship (*IdealCase*). Other factors, including the gender/sexuality of the couple and their relationship type (e.g., monogamous versus non-monogamous) should also be systematically varied.

## Supplementary Material

Supplemental Material

## Footnotes

1 A related literature on oxytocin-enhanced couples therapy is growing in parallel (e.g., Sippel et al. 2023).
