# Supplementary material for: Moral Attitudes Toward Pharmacologically Assisted Couples Therapy: An Experimental Bioethics Study of Real-World “Love Drugs”
Source: AJOB Neurosci. 2024 Oct 18;15(4):239–43. doi: 10.1080/21507740.2024.2402221 (PMC11493051; doi:10.1080/21507740.2024.2402221)

**Supplemental file for**

**Moral attitudes toward pharmacologically assisted couples therapy: an experimental bioethics study of ‘love drugs’**

Mey Bahar Buyukbabani, Brian D. Earp, Ivar Hannikainen, Tommaso Barba,

Emilian Mihailov, David B. Yaden, and Julian Savulescu

**Methods**

Participants were recruited from Prolific and consisted of 288 people based in the UK (*n* = 141 males, *n* = 146 females, *n* = 1 non-binary) with an age range of 21-76 (*M =* 41.4, *SD* = 13.25 ). The original sample consisted of 300 people. The data from 12 participants were excluded due to taking more than two standard deviations above the median completion time to finish the study. Inclusion of participants who took more than two standard deviations to complete the study, did not significantly change the results. Thus only the excluded data was reported.

The study took approximately 4 minutes, and participants were reimbursed 0.45 GBP for their time. The study employed a between-subjects design with three experimental conditions. Participants first completed the consent forms and participant information sheets. They were instructed to carefully read the fictitious scenario on the following page. They were randomly assigned to one of three conditions with different vignettes describing an individual or couple in a long-term romantic relationship considering a treatment to reignite their relationship, and were asked a total of ?? questions about the scenario (see Measures). Finally, participants provided demographic information and completed a comprehension check about the treatment they had just read about.

In all conditions, the treatment involved a pill aimed at reviving romantic love for one's partner by facilitating the appreciation of the partner’s good qualities. However, the stated effectiveness of the treatment varied across conditions. The vignettes varied in whether the described treatment was said to be 100% effective (LantianS2), fairly reliable but not guaranteed (Lantian Realistic), or potentially helpful but requiring additional work, including attending couples therapy from the couple (Ideal Case). See main text Table 1 for the complete wording of the vignettes.

It is important to note that LantianS2 aimed to replicate the design of Lantian and colleagues (2024), who conceptualized the pill as a revolutionary intervention almost akin to a love potion. LantianRealistic was designed to be a more realistic version of the pill that closely mimicked the conceptualization of Earp and Savulescu (2020) of a love drug in their work. Finally, IdealCase aimed to depict an “ideal case” scenario that significantly differed from the other two conditions in various aspects. In this instance, the vignette explicitly mentioned that the couple had kids, similar values, shared goals, and intertwined identities and had already tried numerous relatively conventional methods (e.g., talk therapy, and romantic vacations) to rekindle love. In addition, it was mentioned that this treatment would occur as a part of an intervention done by a prestigious university as a study concerning pharmacologically-assisted psychotherapy for couples.

**Measures**

Participants rated the: (1) perceived realism of the scenario, (2) perceived authenticity, (3) durability, and (4) intensity of the love the couple experienced at the end of the treatment. Moreover, they provided ratings of the (5) perceived morality of the decision to undergo the treatment, the (6) decision makers' morality; (7) willingness to follow, and (8) willingness to allow treatment. All ratings were on 7-point Likert scales with higher ratings representing stronger agreement with the statement. Every dependent variable was assessed with a single item except for authenticity, which was calculated as the average of two items. The wording of all seven dependent measures is provided *verbatim* below:

1. Perceived realism of the scenario
   1. *I find the scenario realistic: (1 = Not At All to 7 = Totally)*
2. Authenticity
3. *Would you say that, at the end of the story, the feelings of love are authentic? (*1 *= Not At All to* 7 *= Totally Agree)*
4. *After the month-long treatment, would you say that the characters really love each other?(*1 *= Not At All to* 7 *= Totally Agree)*
5. Durability
6. In terms of duration, according to you, this love will last: (1= *For A Very Short Time to* 7 *= For A Very Long Time)*
7. Intensity:
   1. *In terms of intensity, according to you, at the end of the story, the feelings of love are: (*1 *= Not At All Intense to* 7*= Very Intense)*
8. Perceived morality of the decision:
   1. *Was it morally justified for the character(s) to decide to undergo the treatment? (*1 *= Not At All to* 7 *= Completely)*
9. Decision makers' morality:
   1. *In your opinion, based on their decision to undergo the treatment, would you say that the individual(s) have a good character, in the moral sense of the word? (*1 *= Not At All to* 7 *= Totally Agree)*
10. Willingness to follow treatment:
    1. *If you were in their position, would you have decided to undergo the treatment? (*1 *= Clearly Not to* 7 *= Clearly Yes )*
11. Willingness to allow treatment:
    1. *If this treatment really existed, should it be banned or should it be allowed? (*1 *= Definitely Banned to* 7 *= Definitely Allowed)*

Further, participants were asked a comprehension question to serve as a manipulation check *“What kind of pill was involved in the treatment that was taken?”* However, no exclusions were made based on this question since we deemed it as potentially too difficult. Participants were also asked about their current relationship status: “*Are you currently engaged in a romantic/intimate relationship?”* and if they ever participated in couples therapy “*Are you currently in or have you ever participated in couples therapy?”* Finally, demographic questions regarding gender, age, level of religiosity, and political orientation regarding economic and social issues were asked.

**Results**

For a visual representation of the results, see the Figures in the main manuscript. Here we report the details of our statistical analyses and tests.

The effect of the condition on each dependent variable was tested using a Kruskal-Wallis test due to our variables being non-normally distributed. Median values were reported alongside 25th - 75th percentile values between parentheses. Significant results were followed up by Dunn's test for multiple comparisons with Benjamini-Hochberg adjustment. Effect sizes were calculated using eta-squared (η²). Please refer to Supplementary Table 1 for a breakdown of descriptive statistics according to condition (i.e., means and respective standard deviations).

***Perceived Realism***There was a significant main effect of condition on the perceived morality of the decision to take the treatment, χ²(2) = 41.58, *p* < .001, and an effect size of η^2^ = .14 (see Figure 1d).

Participants in both LantianS2 [*Mdn:* 2.00 (1.00 - 3.00)] and Lantian Realistic [*Mdn:* 2.00 (1.00 - 3.00)] evaluated the scenario to be less realistic than in IdealCase [*Mdn*: 3.00 (2.00 - 5.00)]. There was no significant difference between the perceived realism of LantianS2 and LantianRealistic (*p* = .453).

***Morality of Decision***
There was a significant main effect of condition on the perceived morality of the decision to take the treatment, χ²(2) = 54.54, *p* < .001, and an effect size of η^2^ = .19 (see Figure 1a).

Participants in both LantianS2 [*Mdn:* 3.00 (2.00 - 5.00)] and LantianRealistic [*Mdn:* 4.00 (3.00 - 5.00)] evaluated the decision to take the treatment to be significantly less moral than in the IdealCase [*Mdn*: 6.00 (4.00 - 7.00)].

Morality ratings of participants in LantianS2 were also significantly lower than LantianRealistic.

***Protagonist Morality***
There was a significant main effect of condition on the perceived morality of the protagonist (i.e., decision-maker), χ²*(*2) = 56.26, p < .001, and an effect size of η^2^ = .19 (see Figure 1a).

Participants in both LantianS2 [*Mdn:* 4.00 (3.00 - 5.00)] (*p* < XXX) and LantianRealistic [*Mdn*: 4.00 (3.00 - 5.00)] evaluated the protagonist as significantly less moral than in the IdealCase [*Mdn:* 6.00 (4.00 - 6.00)].

Moreover, ratings of protagonist morality in LantianS2 and LantianRealistic were not significantly different (*p* = .107).

***Love Authenticity***

There was a significant main effect of condition on the perceived authenticity of love, χ²(2) = 80.28, *p* < .001, and an effect size of η^2^ = .28 (see Figure 1b).

Participants in LantianS2 [*Mdn:* 2.50 (1.50 - 3.00)] evaluated the resulting love to be significantly less authentic than in LantianRealistic [*Mdn:* 3.00 (2.00 - 4.00)] and than in the IdealCase [*Mdn:* 5.00 (3.50 - 5.50)].

Additionally, the IdealCase was significantly higher in authenticity ratings than Lantian Realistic.

***Love Durability***
There was a significant main effect of condition on the perceived durability of love, χ²*(*2) = 21.15, *p* < .001, and an effect size of η^2^ = .07(see Figure 1b).

Participants in both LantianS2 [*Mdn:* 3.00 (2.00 - 4.00)] and LantianRealistic [*Mdn:* 3.00 (2.00 - 4.00)] evaluated the resulting love to be significantly less durable than in the IdealCase [*Mdn:* 4.00 (3.00 - 5.00)].

The durability ratings of LantianS2 and LantianRealistic were not significantly different (*p* = .432.

***Love Intensity***
There was a significant main effect of condition on the perceived intensity of love, χ²*(*2) = 11.21, *p =* .004, and an effect size of η^2^ = .04.

Surprisingly, participants in LantianS2 [*Mdn:* 5.00 (3.00 - 5.00)] and in IdealCase [*Mdn:* 4.00 (4.00 - 5.00)] evaluated the intensity of resulting love to be significantly more intense than in Lantian Realistic [*Mdn:* 4.00 (3.00 - 5.00)].

There was no significant difference between LantianS2 and the IdealCase. (*p* = .192).

***Willingness To Follow Treatment***
There was a significant main effect of condition on participants’ willingness to follow the treatment themselves, χ²*(*2) = 40.91, *p <* .001, and an effect size of η^2^ = .14.

Participants in both LantianS2 [*Mdn:* 2.00 (1.00 - 3.00)]. and LantianRealistic [*Mdn*: 1.00 (1.00 - 4.00)] were significantly less willing to follow the treatment than participants in IdealCase [*Mdn:* 4.00 (2.00 - 6.00)].

Willingness to follow treatment in LantianS2 and LantianRealistic were not significantly different (*p* = .320).

***Willingness To Allow Treatment***
There was a significant main effect of condition on participants’ willingness to allow the treatment, χ²*(*2) = 40.81, *p <* .001, and an effect size of η^2^ = .14.

Participants in the IdealCase [*Mdn:* 5.00 (3.00 - 6.00)] were significantly more willing to allow the treatment than the other two conditions.

Additionally, participants in LantianS2 [*Mdn:* 2.00 (1.00 - 4.00)] showed significantly lower willingness to allow treatment than those in LantianRealistic [*Mdn:* 4.00 (2.00 - 5.00)].

**Supplementary Table 1**

*Means and Standard Deviations of Dependent Variables According to Condition*


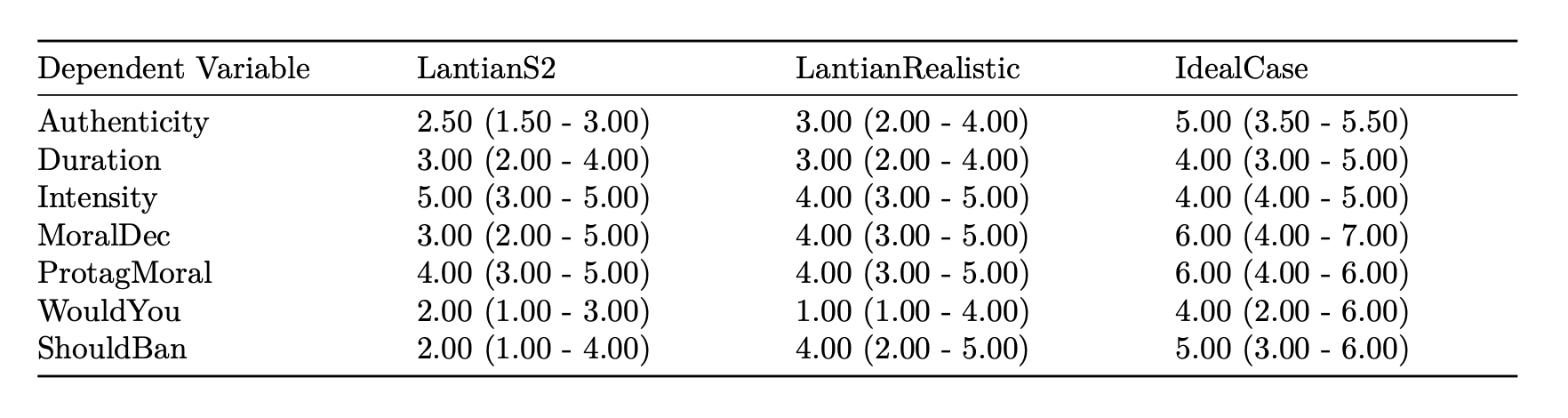

Supplement: Supplemental Material [file UABN_A_2402221_SM5501.docx]
